# The outcomes of Lean management in a large academic hospital in Finland in 2018–2023: a qualitative study

**DOI:** 10.1093/intqhc/mzag079

**Published:** 2026-06-04

**Authors:** Irmeli Hirvelä, Elina Reponen, Mervi Javanainen, Riikka Lämsä, Vesa Niskanen, Paulus Torkki

**Affiliations:** HUS Helsinki University Hospital, Helsinki, Finland; HUS Helsinki University Hospital, Helsinki, Finland; HUS Helsinki University Hospital, Helsinki, Finland; Department of Public Health, University of Helsinki, Helsinki, Finland; Department of Economics & Management, University of Helsinki, Helsinki, Finland; Faculty of Bioeconomy Development, Vytautas Magnus University, Kaunas, Lithuania; Department of Public Health, University of Helsinki, Helsinki, Finland

## Abstract

**Background:**

Lean management (LM) has been implemented in healthcare organizations, promising efficient operations, rapid patient access to care, improved staff satisfaction, and lower costs. However, the use of LM is questioned because there is no strong evidence of its outcomes, so proof of long-term and organization-wide use is needed. The aim of the study is to increase understanding of the long-term effects of LM in healthcare.

**Methods:**

This is a case study done at the Helsinki University Hospital in Finland. A retrospective qualitative study was conducted in three sectors (A, B, and C) of the hospital. Fourteen healthcare leaders from three sectors participated in interviews by answering structured and open-ended questions. The interviews were used to evaluate the outcomes that are experienced by the leaders in the hospital setting over a 5-year period, and evaluate indicators that they used to assess these outcomes. The interviews were analyzed using qualitative and quantitative content analysis with ATLAS.ti analysis software (ATLAS.ti Scientific Software Development GmbH, Berlin, Germany).

**Results:**

The outcomes of LM can be grouped into three main categories (organization, patient, and staff) that are divided into six subcategories: experiential, care-related, work-related, economic, leadership and management, and image effects. The leaders experienced the greatest benefits on the organization level, as it enhanced the effectiveness of the organization’s structure and operations, fostered continuous development, improved staff retention and attraction, and promoted patient-centredness. According to the leaders, the most important benefits for patients included improved access to care, better care flow, enhanced safety of care, and greater participation in improving operations. In addition, the leaders highlighted that the biggest benefits for staff were increased work satisfaction, work competence, work commitment, and ethical behaviour. We found that the use of LM also has disruptive and contradictory effects caused by the lack of organization-wide Lean commitment and structure, and the media have a bias towards negative portrayals of hospital efficiency.

**Conclusion:**

We found that six subcategories provide a comprehensive framework for qualitatively assessing organization-wide experienced outcomes of LM in the hospital. Our findings emphasize that the organization should be fully committed to the Lean method, and its structure should be clear so that the best benefits of LM can be achieved. We noticed that more qualitative LM research is needed to complement the Lean knowledge gained from quantitative research, develop more comprehensive and high-quality evaluation indicators, and increase evidence for its use in healthcare.

## Introduction

In healthcare organizations, adaptability, continuous change, and improvement are important to produce healthcare services that meet the ever-changing needs of the population and the demands of complex health conditions [1, 2]. In a context of continuous change, healthcare organizations should be flexible and provide patients with rapid access to high-quality care at a low cost [2]. These challenges press leaders to look for new ways, and since the 2000s, Lean management (LM) has been one approach to respond to them [2, 3].

Lean is an organization-wide socio-technical management approach [4, 5] that helps organizations deal with uncertainty, diversity, fragmentation, and hierarchy problems; thus, it increases an organization’s adaptative capacity and performance [6–8]. Generally, in hospitals, LM has made operations more efficient and smoother, lowered costs [9, 10], and improved the usability of information technology [8]. International studies have shown that using LM has positive effects on patients: processes are streamlined, waiting times are reduced, and patients are satisfied with their care [7, 11]. As a result of Lean development work, hospital quality of care and safety have increased. For example, infections [12], medical errors [12], and deaths have decreased [12, 13]. Using LM also has positive impacts on employees: their job satisfaction has increased [14], and turnover has decreased in hospitals [2, 7].

Although there are many management studies about LM in healthcare organizations, findings are still contradictory [2, 7, 15–18]. There are only a few organization-wide and long-term implementations of Lean research in healthcare [6, 7, 19]. Also, there is no strong empirical evidence of LM benefits [6] because most of the studies are short-term case studies with weak methodology [6, 19]. Furthermore, there is little information about the failed implementation of Lean and the factors that affect it. It is difficult to compare those findings together because of a lack of a consistent framework for evaluating long-term Lean outcomes. Therefore, from the reviewed literature, we focused on studies related to long-lasting Lean implementation across the healthcare organization.

Recent research findings highlight Lean as a holistic management methodology and continuous improvement system that integrates strategy execution, engages leaders at all levels and develops dynamic learning capabilities—it also serves as an enabler for organizational change [4, 20]. It has also been shown that leaders who are fully committed to Lean and ensure that the organization is focused on it help to facilitate the achievement of the organization’s performance improvement goals, while the absence of leaders’ commitment can hinder performance improvement [4, 21, 22]. For example, over a 4-year period in two Dutch university hospitals, challenges to Lean implementation were a hierarchical organizational structure, siloed operations, and weak interdisciplinary collaboration [20]. These factors led to the failure of Lean implementation due to resistance to change and the lack of a clear strategic roadmap [20].

In healthcare, Lean implementation is a continuing process [23] and varies due to its complexity and the heterogeneity of contexts [6]. However, there has been little research on Lean as a holistic development methodology compared to the use of individual tools, such as visual management or problem-solving tools [24]. The reported improvement results are presented in isolated initiatives without evidence of long-term Lean maintenance [24]. To develop continuous improvement, hospitals should therefore renew themselves culturally and structurally with new visions and goals [24].

There is no unified consensus on how to define success in Lean and evaluate its effects in healthcare [21]. The benefits are often evaluated by process measures, but few cases have reported the health effects on patients [25]. There is also a need to evaluate and develop socio-technical aspects of Lean implementation, practices, and their outcomes [5]. A conceptual framework for classifying outcome measures has been proposed to facilitate benchmarking analyses in Lean healthcare across four main areas: patients, paid and affiliated staff, costs, and service delivery [6].

In this study, our goal is to identify the dimensions of experiential outcomes from the use of LM across the organization. We also attempt to identify indicators that can be used to observe the long-term development of Lean and its results in an organization. We asked two research questions in our study:

RQ1: What kind of experiences do leaders have with Lean management outcomes regarding patients, employees, and the organization?

RQ2: How are these experienced outcomes by the leaders monitored and acted upon in a hospital?

## Materials and Methods

This is a retrospective qualitative study in which we aim to understand the Lean phenomenon under research from the perspective of participants and to describe how they explain their experiences in 2018–2023 [26]. Because the study was conducted at a single academic medical centre, it can be called a case study, which provides more in-depth information into the real context of a phenomenon [27]. The aim of this study is to evaluate the use of LM and deepen the understanding of the Lean philosophy, both related phenomena and dimensions, in the healthcare context. We have used COREQ qualitative reporting standards for this study [28].

### Research settings

The research involves a case study done at the large academic medical centre, Helsinki University Hospital (HUS) in Finland. The Finnish healthcare system is mostly a tax-based, Beveridge-type healthcare system model in which the government provides healthcare for all its citizens, and according to this, HUS is also mainly publicly organized and maintained with tax revenue.

HUS is the largest provider of specialized healthcare in Finland and employs over 27 000 multi-professional workers. Every year, approximately 680 000 people are treated at the hospital. The hospital has been using the Lean method since 2009, and there is a separate Lean unit where trainers help to develop the hospital’s operations. Lean methodology has been applied at a strategic level in the research hospital for an extended period. However, due to the organization’s large size and complexity, implementation has progressed at varying paces across different sectors, with model cells serving as a mechanism to support and guide the adoption process. Only a limited number of studies on LM have been conducted in this hospital [4, 6, 29, 30].

This study evaluated three hospital sectors (A, B, and C), which were selected on a voluntary basis and were broadly representative of the hospital’s operations. The sector A includes mainly daytime outpatient clinics and elective surgical care, the sector B includes inpatient wards, outpatient clinics, and emergency care, and the sector C provides elective and emergency surgery care. Sectors A, B, and C are described in Table 1.

**Table 1:** Employees and treated patients in 2023.

Year 2023	Sector A	Sector B	Sector C
Employees	802	1169	1215
Treated patients in the own sector	123 154	192 938	54 144
Treated patients in purchased services	50 484		
Treated patients in other sectors			86 854

### Data collection and analysis

We collected data through structured and open-ended questions in focused interviews in a single academic medical centre. All data were collected during 2024 and analyzed in 2025. First, the researcher (IH) presented the study at three sector meetings, where the leaders were briefed on the study. After this, the sector leaders provided the names of their subordinates’ leaders to the researcher, who then contacted 17 leaders by email in random order. From contacted 17 leaders from three sectors, 3 of whom declined, citing their busy working schedules, and 14 voluntarily agreed to be interviewed. The researcher arranged interviews with the volunteer leaders, either face-to-face or online, according to the leaders’ wishes. One researcher (IH), who has trained in Lean methodology and has 7 years of experience as a Lean coach, interviewed all participants and collected the data. The language that was used in interviews was the native language of the leaders and the interviewer. All data were translated by the interviewer (IH). Themes of the interviews were the background of the leader, indicators that are used to follow outcomes in a unit/sector/division, and developing actions and their outcomes for patients, staff, and the organization between the years 2018 and 2023 using LM.

The 14-volunteer middle management and director-level healthcare leaders interviewed were leading the units/divisions/sectors in the selected three sectors. Thirteen (13) of the 14 leaders were experienced using LM and trained in Lean methodology. The leaders’ experience using LM in their work ranged from 1 to 14 years. The 14 leaders received the interview questions in advance (Attachment 1), and they signed an informed consent-to-be-interviewed form before the interview. We used structured interviews, in which the interviewer asked the interview questions and clarified the questions as needed. The interviews averaged 1 h in duration and were recorded and transcribed. The number of interviews was interpreted to be sufficient as the responses started to become saturated. The total of transcribed pages was 127.

At first, we analyzed the interviews using inductive qualitative content analysis [26, 31] with ATLAS.ti analysis software (ATLAS.ti Scientific Software Development GmbH, Berlin, Germany). We used inductive content analysis for data processing because it is suitable for analyzing complex, sensitive phenomena in healthcare [31]. Our goal was to obtain a comprehensive description of the LM phenomenon and to identify categories that describe it [25].

Data were transferred to the ATLAS.ti analysis software, and relevant quotes from the text were coded to 157 different codes. These codes were related to quotations of data 462 times. After that, we used methodological triangulation to confirm and validate our findings; both qualitative and quantitative content analysis were used for data [27, 32]. In the quantitative content analysis, we used frequencies (how often a word, phrase, or theme occurs) to identify codes, subcategories, and categories. In addition, qualitative content analysis (data coding) was used to identify the external and internal meanings of the categories [32]. The parallelism of quantitative and qualitative aspects facilitated content analysis, as well as demonstrating the conclusions of the results [27, 32]. In the review, the codes were renamed, combined, and divided, and findings were discussed with the research group. The analysis continued with the abstraction of the codes to the six (6) subcategories, i.e. finding connections and interconnections between the codes. Finally, the subcategories were divided into three (3) main categories: outcomes of the organization, patients, and staff.

In our findings, we use a single term—leader—to describe a leader of the unit/sector or division in the hospital. We present our findings as numbers, where the first number is how many times the code appears in the data, and the second number represents the number of leaders who bring up this code.

## Results

**Figure 1:**
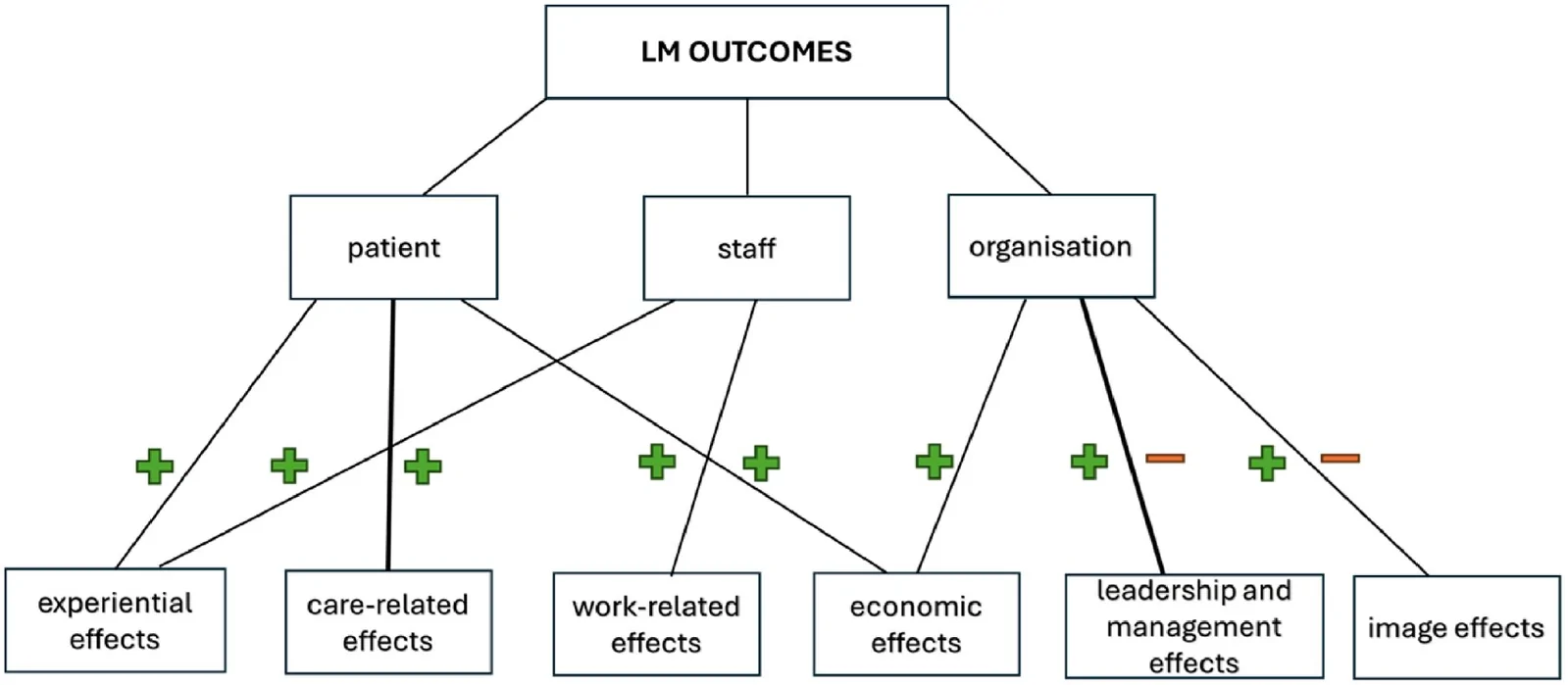
The outcomes of LM.

We present our composite findings in Fig. 1. Our findings were divided into six (6) subcategories: experiential, care-related, work-related, economic, leadership and management, and image effects, which formed three (3) main categories (organization, patient, and staff). These six subcategories have positive and/or negative effects on the main categories. The strongest positive effects were between the main category—organization and the subcategories—leadership and management. And other stronger positive effects were between the main category—patients and the subcategory—care-related effects. On the other hand, the negative effects were between the main category organization and subcategories—leadership and management, and image effects.

In this results section, the effects of LM were grouped into positive and negative effects that the leaders expressed. Positive effects were the benefits that LM brought to the organization, patients, and staff. Negative effects were grouped into either disruptive or conflicting effects by their nature. Disruptive effects prevented the benefits of LM from being achieved, and conflicting effects turned the benefits of LM into opposite, negative effects on the organization, patients, and staff. In addition, we present the indicators that leaders used to measure the outcomes of LM.

### Benefits of using Lean management

The leaders emphasized that the benefits of using LM were greater for the organization (80 quotes/14 leaders) than for patients and staff. These benefits were management and leadership, economic, and image effects (Table 2). The management and leadership benefits (75/14) were that the management structure of the organization becomes clearer and more effective (34/12), and a continuous development increase (28/8). The leaders highlighted that the use of LM makes operations logical, structured, and easily manageable. A layered and low-level management structure enabled rapid response to various needs. They stated that continuous development with staff made operations more efficient and streamlined patient flow.

I would say that it is [a management structure]. It is structured, logical, easy to manage. It is quite easy to set goals for each X and to move things forward and develop. I would say that it is, overall manageability, right away. Now, it exists. (Leader B1)

**Table 2:** The LM outcomes for the organization.

Positive outcomes: 80 quotes/14 leaders
**1. Management and leadership effects: 75/14** **1.1. Clearer management structure: 34/12** • Operations are logical, structured and easy to manage; things are moving forward together with staff, and development is continuous • By developing operations and eliminating waste, the units’ operations become more efficient • Smooth flow of patients • Unnecessary cancellations have been eliminated by thinking about operations *I would say that it [a management structure] is. It is structured, logical, easy to manage. It is quite easy to set goals for each X and to move things forward and develop. I would say that it is, overall manageability, right away. Now, it exists. (Leader B1)* **1.2. Continuous development increases: 28/8** • The culture has changed; the readiness to try new things and stick with them increases • Sharing best practices with other units has increased *It is the kind of thing where we have to repeatedly remind ourselves that that is why the data are collected and that is why we try to notice the deviations that could be addressed, and that the creation of that culture is still in progress—we are not there yet. We may now recognise the challenges, but we need to take development actions, and there is still work to be done. (Leader A1)* **1.3. Staff retention and attraction increase: 11/6** • Personnel are satisfied with their work and remain in the units • The workload of specialist doctors becomes more rational *Well-being at work improves, so you could think that the retention of personnel improves and through that we also save on orientation costs. (Leader C1)* **1.4. Patient-centeredness increases: 2/2** *We have a lot of changes that have made our operations more patient friendly. For example, it [a centralized callback service] was good, so it’s back. Phone issues, and then there are many other things like that. I even mentioned the efficiency of getting these electronic patient forms to meet our needs. (Leader B3)*
**2. Economic effects: 3/3** • Saves on various costs, such as personnel and supplies *For example, in that X process, from the point of view of the entire organisation, money should be saved. That is annoying because inpatient resources for it are smaller. But of course, I don’t know if that money has been spent on something else in the organisation. (Leader C4)*
**3. Image effects: 2/2** • High quality of care improves the hospital’s image both nationally and internationally *It’s great that you’ve been so successful nationally and then internationally. (Leader B4)*

According to the leaders, using LM staff retention and attraction (11/6) at the organization improves and patient-centredness (2/2) increases. They described that staff’s opportunity to influence daily work increased their loyalty to the organization. They also pointed out how daily meetings and operational development, in line with Lean thinking, focused on patients and their care.

We have a lot of changes that have made our operations more patient friendly. For example, it [a centralized callback service] was good, so it’s back. Phone issues, and then there are many other things like that. I even mentioned the efficiency of getting these electronic patient forms to meet our needs. (Leader B3)

Financial benefits (3/3) and image effects (2/2) were also mentioned. Financial effects (3/3) were savings on supplies and personnel costs, and positive image (2/2) effects. The positive image effects were like successes outside the organization, such as lower infection statistics after surgeries in international comparisons.

The perceived benefits for the patients (60/14) were experiential, care-related, and economic effects (Table 3). The leaders most emphasized (49/14) care-related effects for patients, such as improved access to care, care flow, and safety of care. In addition, the patients’ participation in the improvement of operations increased. For example, representatives of patient groups were involved in developing a new hospital building to ensure their specific needs were considered.

Of course, patients get treatment faster. That’s probably the goal of many projects. Patient satisfaction, like influencing that in general. Well, if it’s about patient satisfaction, usually we have very satisfied patients, and then those dissatisfactions are most often related to access to treatment, so I think that’s probably the most important thing. (Leader A4)

**Table 3:** The LM outcomes for patients.

Positive outcomes: 60 quotes/14 leaders
**1. Experiential effects: 6/6** **1.1. Satisfaction increases** • Satisfaction of care increases *Our patient satisfaction is really high. We have that in emergency rooms, too. So that’s constantly at 90/100. (Leader B3)*
**2. Care-related effects: 49/14** **2.1. Access to care improves: 20/14** • Access to care is faster • Patients have more direct contact with the professionals treating them • Patients are contacted more lightly before procedures, e.g. by telephone and through electronic systems *Of course, patients get treatment faster. That’s probably the goal of many projects. Patient satisfaction, like influencing that in general. Well, if it’s about patient satisfaction, usually we have very satisfied patients, and then those dissatisfactions are most often related to access to treatment, so I think that’s probably the most important thing. (Leader A4)* **2.2. Care flow improves: 20/14** • Patient throughput of care is more efficient • Forecasting and planning of units’ operations increase, e.g. trying to avoid sudden cancellations • Daily management meetings bring planning to patient care and speed up their discharge from units to home • A patient meets with several professionals during one visit at a hospital • Patient segmentation, e.g. more personalised care for patients • Only high-risk patients come to pre-visits before operations • Patients receive the right treatment at the right time *And then, yes, we have been able to streamline operations, and through that, we have been able to operate on more patients, that it appears to the patient so that they can undergo surgery. (Leader C1)* **2.3. Safety of care increases: 3/3** *Patient safety work has been able to increase safety for an individual patient. Of course, the patient does not, that is when nothing happens. What is the positive manifestation of safety, that fact, then the patient does not know that something negative could have happened. (Leader C1)* **2.4. Participation in operations improvement increases: 6/6** • The units’ operations renew according to patient feedback *Following patient feedback as part of this daily management has also brought about development targets and insights from the patient’s perspective that have been taken forward. (Leader A1)*
**3. Economic effects: 5/3** **3.1. Medical expenses decrease: 2/2** • Treatment and care costs are reduced **3.2. Sick leaves and the resulting reduction in income decreases: 3/3** • Patients return to work faster *There are also such patients when they go home, so their sick leave is also shorter, and they can get back to work without such cost impacts. The thing is definitely a cost impact, yes that it is. After all, it is then a shorter sick leave. (Leader B2)*

The experiential effects were the next most mentioned (6/6). According to six leaders, the patients’ satisfaction indicators of care were increased. In addition, economic effects on patients were highlighted (3/3)—their medical expenses, sick leaves, and the resulting loss of income were reduced while the hospital’s operations were continuously improved. During the interviews, the leaders did not mention any negative effects on patients.

The benefits of LM for the staff (31/14) were mentioned less often. These were experiential and work-related effects (Table 4). According to the leaders, LM had the greatest effect on the staff’s work (28/14). These effects were increased work satisfaction, work competence, work commitment, and good ethical behaviour. During the interviews, the leaders presented benefits of the staff’s commitment to their work, such as an increase in genuine listening of the staff, multi-professional cooperation, transparency towards staff, a smooth workflow and its importance to the staff, and an increase in the attraction and retention of the staff. According to the leaders, personnel indicators showed that the staff’s job satisfaction has increased and staff turnover has decreased. Additionally, they said that the staff described the operations of units as ethically sustainable.

**Table 4:** The LM outcomes for the staff.

Positive outcomes: 31 quotes/14 leaders
**1. Experiential effects: 3/3** **1.1. Appreciation increases** • Staff feel like they are being heard and can participate • Public rewards after staff development *The fact that someone feels heard and gets to participate in it [a development of unit]—yes, when they have joined in, I feel that it has increased satisfaction. (Leader B4)*
**2. Work-related effects: 28/14** **2.1. Job satisfaction increases: 4/4** • Meaningfulness of work increases *If we look at the personnel surveys that come out once a year, and just a moment ago, we checked the results of the last survey within the hospital, they seem to be improving, all the indicators there are still a lot of things to do. And the indicators are not really, they are all still, but at least in particular which ones were the lowest, the worst, or I guess all the indicators have turned in a more positive direction. (Leader C2)* **2.2. Competence of work increases: 6/3** • Knowledge and skills of work increases • Quality of work increases *It’s like people have brains that they can think all the time—let’s develop together. (Leader A2)* **2.3. Commitment to work increases: 16/14** • Bringing forward development ideas and trying them out with a low threshold are allowed • Staff are committed to their work and ready to help others • Staff are involved in the change • Dialogical interaction increases • Transparency towards staff increases • Smoothness of work increases • Job retention and attractiveness increases *The staff also become more committed when the problem we have had becomes visible; that is, the purpose was that people commit better when they know about it [the problem]. And when they see that there is a shortage of personnel somewhere or that help is needed somehow, then they still have it or when they are more ready to go and help you. But if you don’t talk now, if you don’t know anything about the situation, then you can’t really commit, that it is also like a person’s time. At the beginning, no one believed that because it was considered just like this, as an end in itself and just crunching numbers and so on. But when the team started to understand, then when they knew how many operations there were per day, then that’s when the situation started to change. Before, no one knew when, there were just people who operated then, and it was a black hole. (Leader B5)* *Yes, it is also such a holding and attraction force, like in terms of the staff, that it is somehow meaningful. It brings such meaningfulness that we have different, in a way, patient care paths. (Leader B2)* **2.3.1. Multi-professional cooperation increases: 2/2** • Increased understanding of the work done by others *Such as understanding what other people’s tasks are related to and in a way such as a slap in the face that you just can’t do it faster or something. If you don’t really understand what the other person is doing and what they are responsible for, I would imagine. That’s right. Such a really multiprofessional collaboration moves a little, a little more in those, and it comes in a way that when your eyes open when others describe what they are doing during a process. That is, it improves multiprofessional collaboration and understanding in a way. (Leader B3)* **2.3.2. Ethical behaviour at work increases: 2/2** • An ethical work culture increase *Especially the fact that from unit X, those private clinics bought nurses and doctors with money at one time, but many of them have returned, and they feel that in the public sector, it is ethical to operate and to do good quality work, and then the job descriptions are more diverse. (Leader B2)*

The experiential effects (3/3) included an increase in staff appreciation. Three leaders said staff expressed that they felt heard and valued in their work when they actively participated in the unit’s operation and improvement. The leaders did not mention any negative outcome of LM on staff during the interviews.

The staff also become more committed when the problem we have had becomes visible; that is, the purpose was that people commit better when they know about it [the problem]. And when they see that there is a shortage of personnel somewhere or that help is needed somehow, then they still have it or when they are more ready to go and help you. But if you don’t talk now, if you don’t know anything about the situation, then you can’t really commit, that it is also like a person’s time. At the beginning, no one believed that because it was considered just like this, as an end in itself and just crunching numbers and so on. But when the team started to understand, then when they knew how many operations there were per day, then that’s when the situation started to change. Before, no one knew when, there were just people who operated then, and it was a black hole. (Leader B5)

### Disruptive and contradictory effects with the use of Lean management

In the interviews, 13 of the 14 leaders pointed out some disruptive and contradictory effects with the use of LM. The disruptive effects (13/13) were a lack of an organization-wide commitment to Lean and a lack of management structures above the divisions and sectors. They also pointed out that the organization’s goals were complex and not measurable. These were significant disruptive factors and bottlenecks that hindered the effectiveness of organization-wide management and operation, as well implementation of Lean at the hospital level.

In my opinion, if we think that we want to preserve the benefits that have already been achieved. And the transformation, which is a long process, and we are clearly in the middle of it [a Lean implementation] in all sectors, but especially now we are talking about the x centre, then we should then, up to the senior management and probably the senior management means the CEO in this point, commit to this [LM] and communicate that commitment quite strongly. And really, in a way, to prevent the deterioration of the things that have already been achieved and to keep the wheel moving in the direction that we want. That there should be a common understanding of what is wanted. (Leader C1)

One contradictory effect (2/2) on the organization was the negative image effect by the media. Two of 14 leaders mentioned that when an organization improves its operations, the media often portrays it negatively, which can create fear of healthcare among patients.

I was also left thinking about that, that actually, the damage to its reputation can be very great and then it becomes like that, like that image stays alive and that if someone starts to think, even though they will definitely think through these newspaper articles, that they shouldn’t go to clinic X at all. So somehow it is painted as if we are big, and everyone is treated the same. That is not true. In my opinion, the media has a tremendous amount of power. (Leader B2)

### The indicators of Lean management outcomes experienced by the leaders

The leaders (41/14) brought up the usability of LM outcome indicators. The followed LM outcomes were evaluated by using the LM methodology named Hoshin Kanri, so-called catchball method [33] from the organizational strategy to the individual division, sector, and unit level. Some of the indicators to be monitored were a result of Kaizen events [20] and as development suggestions brought by the detection of problems and patient and staff feedback.

The main LM outcome indicator that every leader monitored daily was access to care (care-related), such as patient volumes and factors related to the smooth flow of patient care (e.g. operating room starts and changeover times). Additional care-related indicators were followed weekly and monthly, such as performance indicators and referral processing times, as well as outpatient clinic, operating room, and inpatient ward utilization and occupancy rates. The leaders also followed other LM care- and work-related and financial effects on patients, staff, and the organizations (e.g. cancelled outpatient clinic visits and operations, staff absences, infection rates, adverse event rates, patient satisfaction, staff job satisfaction, and economic indicators). Operational indicators and issues related to the effectiveness and smoothness of the day’s work were monitored in daily meetings with staff using electronic boards or whiteboards.

The leaders (34/14) raised several problems related to LM outcome indicators. The problems with indicators were that they were not necessarily developed to meet management needs. There was a lack of qualitative assessment and indicator-based information—not all indicators were available electronically in real time. For example, regarding the LM work-related effects on staff, the leaders were aware of staff turnover based on available staff data, but job satisfaction was measured only once a year. This presented a challenge to respond flexibly to problems and challenges related to staff well-being at work. The hospital also lacked other work-related organizational indicators, such as predictability of future performance and operations.

The indicators of daily available resources in the hospital (facilities and staff) were lacking, which led to waste and hindered operations. The leaders also mentioned that patient classification and measures for cost-effectiveness of patient care should be developed and utilized more. The LM benefits of management and leadership were only monitored with outcome indicators about patients. The organization has not considered or assessed the overall effects of clear management structures, effective operations, and the experiential effects of LM on patients and staff, nor patient-centredness or image effects.

The leaders (28/14) expressed the usefulness of LM care- and work-related indicators. The indicators had been utilized in managing and streamlining operations, solving problems, continuously improving operations, increasing multidisciplinary cooperation, justifying additional resources (facilities, equipment, and personnel), and responding to internal and external change factors in the hospital.

It [LM] has become part of our operating culture that we do this and that. Although, I am afraid that if someone is completely new in top management, it will be seen that something has gone wrong. Yes, it [a new leader] will suddenly start to deteriorate, but that it [LM] is in a way that the responsibility for identifying problems or such poorly functioning process points and doing something about them is there. That is how it [Lean] is mentally in the unit, and not that it has to be dictated from above, that it is more of a coaching discussion, that hey, that the option has been there for a couple of days now. It is or longer, that is there a reporting problem here or what is it about, that is. That my job is to throw the ball, but not to say that now you will do this or that sometimes to them. (Leader C1)

## Discussion

### Statement of principal findings

In healthcare, the complexity of Lean implementation contexts and the heterogeneity of the environment make it difficult to evaluate LM outcomes [4]. According to our results, a minimum period of 5 years into the LM implementation is suggested when evaluating the benefits of LM comprehensively. The management and leadership benefits were highlighted. A clear low-level management structure enables an effective and rapid change of operations. Oppositely, the significant disruptive effect of LM was the lack of commitment to Lean and the missing organizational structures. In other words, the partial Lean implementation acts as a counter-effect to operations of the organization and its continuous development. All these findings extend previous systematic reviews [6, 8, 19], where the findings are presented as individual development projects without long-term Lean maintenance and organization-wide results.

Based on our findings, the effects of LM extend to the entire healthcare system, hospitals, their operations and external environments, including the people who are part of it—employees and patients. For example, the number of patients in the primary healthcare is increasingly affected if patients’ access to hospital treatment is delayed. Conversely, if patients can return to work more easily after surgery, it benefits the employers, families, work communities, and the society as a whole.

The effects of LM can be categorized into experiential, care- and work-related, financial, management and leadership, and image-related effects. Earlier research also mentions several outcomes that are not easily measurable, such as a clearer organizational focus or better multi-professional collaboration [4, 21], but organization-wide information based on qualitative assessment and their indicators has not been presented before. The previous systematic review findings categorize the outcomes into four indicators: (i) patients, with a subcategory of patient experiences, (ii) paid and affiliated staff, (iii) costs, and (iv) service provision [6], and we extend this by adding one main indicator—(v) image—and lifting (vi) experiences of patients and staff into main indicators. Therefore, within measuring quantitative outcomes, it is also important to qualitatively assess the outcomes of LM to have a more comprehensive and deeper understanding of the effects of LM, to recognize potential mechanisms that lead to measurable outcomes.

Some of our findings align with the previous studies that illustrate similarities in the benefits and barriers to LM use. The positive benefits of using LM include time savings [7, 9, 11, 24], reduced errors [8, 12, 24], identification and reduction of waste [8, 9, 24], reduced costs [7–10, 24], identification of bottlenecks [9, 24], increased efficiency and productivity [7, 8, 24], improved patient flow [8, 9, 24], positive impact on quality and safety of care [7, 24], and improved staff and patient satisfaction [7–9, 11, 24]. The examples of barriers faced in organizations are low participation and commitment to Lean [4, 17, 21, 24], difficulties in collecting the necessary data [21, 24], failed communications [21, 24], and excessive bureaucracy for systematic change [17, 24]. The similarities regarding these findings make this research transferable and relevant for qualitatively evaluating LM outcomes in other healthcare contexts.

### Interpretation within the context of the wider literature

Evidence on the usefulness of using LM in healthcare organizations is conflicted, and international research shows that the field of Lean research is narrow and siloed. One possible explanation for the differences in the results of LM outcomes is the heterogeneity of research contexts. According to our findings, the disruptive effects and the contradictory effects can be seen as endogenous and exogenous factors in accordance with organizational theory [34]. We noticed that contexts are endogenous factors or characteristics that exist within organizations, and individuals participating in the Lean method are influenced by exogenous factors. A deeper understanding of these things can help in the implementation of Lean and the optimization of its results in different organizations.

We agree with the wider research that Lean is a socio-technical management method [4–6, 16, 17, 20–22, 24], and when implementing LM in a service sector, such as healthcare, the human perspective should be considered, and experiential outcomes are significant parts when evaluating the LM outcomes. Our findings show that continuous evaluation of LM outcomes increases the understanding of Lean in healthcare and challenges it to expand its theoretical framework regarding service sectors, healthcare.

### Strengths and limitations

The strength of this study is the application of a qualitative content analysis together with quantitative methods, which is less frequently used in LM research, in the healthcare context. Additionally, the strength of this study is that the findings and their validity were discussed among the research team by reviewing the data and their interpretations several times, so they were verified. Our findings show that more descriptive information is needed to support the quantitative data to gain an in-depth understanding of the outcomes of LM.

The limitations of this study are that there was only one researcher who interviewed all participants, meaning the researcher’s objectivity and knowledge about Lean should be considered with the findings, although all the interview questions were structured to minimize bias. Interpersonal and contextual factors also emerged when we compared face-to-face and online interviews that the researcher tried to avoid with objectivity, pre-defined questions and, where necessary, clarifying questions.

The limitations of this research are that the conducted interviews did not cover every leader of sectors in the hospital, and the number of participants was limited (*N* = 14). However, during the interviews, the responses became saturated, so the data was sufficient.

For further research, it would be good to increase the use of qualitative research on Lean to obtain more information about it. Especially, hospital outcome measures can be affected by several confounding factors, so more quantitative research is needed to link qualitative findings with the results of Lean implementation and how Lean appears to organizations, patients, and staff. It is also needed to develop indicators that assess organization-wide outcomes of LM, such as experience-based outcomes, and predictive models of LM to support healthcare management.

### Implications for policy, practice, and research

In Finland, the healthcare financing system changed in 2023 to a fixed budget instead of the earlier activity-based reimbursement [35]. This has put pressure on leaders regarding the organization of patient care and the cost-effectiveness of operations. Our research brings up the financial benefits of using LM for both the organization and patients reported by the leaders we interviewed, and these findings are consistent with those published in previous literature [7–10, 24]. However, the economic benefits for patients have been less studied. In the face of significant cost-containment pressures in healthcare organizations, it remains important to prioritize long-term cost-effectiveness as a guiding principle in service development and operational planning, while also considering the economic impact on patients.

The visions and strategies of organizations should be easily implemented and measurable so that they can be followed and operations can be continuously improved [4, 6, 9, 24]. The organizational development may be difficult to measure by quantitative indicators. Therefore, the qualitative assessment and its indicators may be essential to identify whether the operations are improving in the targeted direction.

In this research hospital, previous studies have predominantly examined the use of LM from a quantitative perspective and have often been confined to a specific time frame [29, 30]. These limitations highlight the need for longitudinal, hospital-wide studies that observe not only the implementation of LM but also its sustained use over time. Furthermore, a mixed-methods approach combining qualitative and quantitative research is needed to achieve a deeper and more comprehensive understanding of how LM is adopted, embedded, and sustained in practice.

From a research perspective, a commonly used, organization-wide Lean outcomes framework that includes qualitative aspects has been lacking. Our results show that the use of six subcategories in research could provide additional information on long-term results across the organization. Using this framework in research would enable international comparison of the outcomes of Lean initiatives and strengthen LM use. It would also provide policymakers and leaders with stronger evidence for LM in healthcare.

## Conclusion

This study shows that qualitative, experiential outcomes and their indicators are needed to complement quantitative research when assessing the organization-wide and long-term effects of LM in healthcare. The main theoretical finding is that the use of LM has experiential, care-related, work-related, financial, leadership and management, and image-related effects. Evaluating these effects enables a more comprehensive understanding of LM. From a practical perspective, our study shows that organization-wide commitment to and implementation of Lean are key elements in achieving its potential benefits in healthcare.

## Supplementary Material

mzag079_Supplementary_Data

## Data Availability

Data are available only on request.

## Permission

HUS Helsinki University Hospital.
