# Supplementary material for: The outcomes of Lean management in a large academic hospital in Finland in 2018–2023: a qualitative study
Source: Int J Qual Health Care. 2026 Jun 4;38(3):mzag079. doi: 10.1093/intqhc/mzag079 (PMC13345745; doi:10.1093/intqhc/mzag079)
Supplement: mzag079_Supplementary_Data [file mzag079_supplementary_data.zip › Attachment 1. Interview Questions for the Leaders.docx]

**Attachment 1. Interview Questions for the Leaders**

**Background question**

1. Are you a leader of a unit, division or sector?

**Lean knowledge**

1. Have you completed the Lean training? NO=0, YES=1
2. Did someone prepare you for the Lean training? NO=0, YES=1
3. How long have you used Lean in your work (in years)?
4. Evaluate your knowledge regarding the use of Lean management in your leadership. Use the five-point scale in your evaluation (Very poor=1, Poor=2, Fair=3, Good=4, Very good=5).
5. Evaluate your commitment to Lean management and development in your unit/division/sector. Use the five-point scale in your evaluation (Very poor=1, Poor=2, Fair=3, Good=4, Very good=5).
6. How many Lean trainers do you have in your sector overall?
7. Do you use the layered management system? NO=1, PARTIALLY=2, YES=3
8. Do you use the daily management system? NO=1, PARTIALLY=2, YES=3
9. Have the staff in your unit/division/sector received the Lean training? NO=1, PARTIALLY=2, YES=3
10. Did someone prepare them for the training? NO=1, PARTIALLY=2, YES=3
11. Does the staff receive continuous Lean training? One-time training=1, Occasional training=2, Continuous training=3
12. Evaluate the Lean knowledge of your staff. Use the five-point scale in your evaluation (Very poor=1, Poor=2, Fair=3, Good=4, Very good=5).
13. What Lean tools do you use in your leadership management and development in your unit/division/sector?

Lean tools:

1. 5S, NO=0, YES=1
2. A3, NO=0, YES=1
3. Value stream map/value stream mapping, NO=0, YES=1
4. Kanban-board, NO=0, YES=1
5. PDCA, NO=0, YES=1
6. Gemba, NO=0, YES=1
7. Kaizen, NO=0, YES=1
8. Other (describe what)
9. What part of your staff understands the goals of the daily operations? None=1, Some=2, Most=3, All=4
10. Evaluate your staff’s Lean commitment. Use the five-point scale in your evaluation (Very poor=1, Poor=2, Fair=3, Good=4, Very good=5).

**Indicators**

1. What indicators do you use in daily operations in your unit/division/sector?
2. Why did you choose these indicators?
3. Where did the indicators that you use come from?
4. Describe, how do you use these indicators in your work?
5. How often are these indicators measured? Occasionally=1, Monthly=2, Weekly=3, Daily/More than daily=4
6. How easy is it to get relevant results from these indicators? Use the five-point scale in your evaluation (Very poor=1, Poor=2, Fair=3, Good=4, Very good=5).
7. Do these indicators convey enough information? Use the five-point scale in your evaluation (Very poor=1, Poor=2, Fair=3, Good=4, Very good=5).
8. What other indicators would you like to implement and why?

**Development**

1. How has your unit/division/sector developed throughout the years 2018–2023? What were the main goals?
2. What Lean tools and methods have you used in these developments?
3. How have you succeeded in implementing the continuous development process? Use the five-point scale in your evaluation (Very poor=1, Poor=2, Fair=3, Good=4, Very good=5).
4. Has your unit/division/sector had ‘breakthrough projects’? If so, describe them.
5. Was there any outside influence affecting your unit/division/sector in the years 2018–2023? If so, describe them.
6. How did these outside influences affect your daily operations?
7. Has Lean management been used to combat any challenges faced in development?
8. Describe, how does the development affect the patients?
9. Describe, how does the development affect the staff?
10. Describe, how does the development affect the organisation as a whole?
